# Initial Muscle Quality Affects Individual Responsiveness of Interleukin-6 and Creatine Kinase following Acute Eccentric Exercise in Sedentary Obese Older Women

**DOI:** 10.3390/biology11040537

**Published:** 2022-03-31

**Authors:** Ivo Vieira de Sousa Neto, Dahan da Cunha Nascimento, Jonato Prestes, Eduardo Fernandes da Fonseca, Rodrigo Souza Celes, Nicholas Rolnick, Yuri Gustavo de Sousa Barbalho, Alessandro de Oliveira Silva, Marina Morato Stival, Luciano Ramos de Lima, Silvana Schwerz Funghetto

**Affiliations:** 1 Laboratory of Molecular Analysis, Faculty of Ceilândia, Universidade de Brasília, Brasilia 70910-900, Brazil; 2 Graduate Program of Sciences and Technology of Health, Faculty of Ceilândia, Universidade de Brasília, Brasilia 70910-900, Brazil; yurigustavo.sousa@gmail.com (Y.G.d.S.B.); marinamorato@unb.br (M.M.S.); silvana.funghetto@gmail.com (S.S.F.); 3 Graduate Program of Physical Education, Catholic University of Brasilia, Brasilia 70910-900, Brazil; dahanc@hotmail.com (D.d.C.N.); jonatop@gmail.com (J.P.); eduardofernandesdafonseca@gmail.com (E.F.d.F.); celes.rodrigo@gmail.com (R.S.C.); 4 The Human Performance Mechanic, Lehman College, New York, NY 10468, USA; nick@thebfrpros.com; 5 Faculty of Physical Education and Medicine, Center University of Brasilia, Brasilia 70910-900, Brazil; silva.alessandro.oliveira@gmail.com; 6 Department of Nursing, Faculty of Ceilândia, Universidade de Brasília, Brasilia 70910-900, Brazil; enframosll@gmail.com

**Keywords:** cytokines, muscle, physical activity, eccentric training, responders

## Abstract

**Simple Summary:**

Muscle health should be prioritized in individuals with conditions who may be at risk of obesity accompanied by sarcopenia. In this context, muscle quality is a novel index of functional capacity that is increasingly relied upon as a critical biomarker of muscle health in low-functioning aging. However, there is scarce evidence regarding which muscle phenotype elicits a more robust effect on important molecules responsible for muscle regeneration and energy homeostasis. Hence, in this study, we evaluated the time-course responses on interleukin-6 and creatine kinase levels following acute eccentric resistance exercise in sedentary obese elderly women with muscle quality differences. This issue is valuable because eccentric exercise may be a particularly promising approach for older adults to efficiently improve muscle mass, strength, and functional performance. We observed that obese older women with high baseline muscle quality display significant increases of interleukin-6 and creatine kinase following an acute eccentric exercise intervention. Such individuals are commonly termed “high-responders”. In contrast, the participants with low muscle quality showed exceptionally small responses (“low responders”) for these molecules compared to individuals with high baseline muscle quality. Muscle phenotype can potentially contribute to individual variation in exercise responses. This phenomenon of responsiveness may clarify important training adaptations.

**Abstract:**

This study aimed to evaluate the time course and responsiveness of plasma interleukin-6 (IL-6) and creatine kinase (CK) levels following acute eccentric resistance exercise in sedentary obese older women with a different muscle quality index (MQI). Eighty-eight participants (69.4 ± 6.06 years) completed an acute eccentric resistance exercise (7 sets of 10 repetitions at 110% of 10-repetition maximum with 3 min rest interval). Participants were divided into two groups: high or low MQI according to 50th percentile cut-off. The responsiveness was based on minimal clinical important difference. There were no differences between groups and time on IL-6 and CK levels (*p* > 0.05). However, the high MQI group displayed a lower proportion of low responders (1 for laboratory and 2 for field-based vs. 5 and 4) and a higher proportion of high responders for IL-6 (7 for laboratory and 6 for field-based vs. 4 and 5) compared to low MQI group. In addition, the high MQI group showed a higher proportion of high responders for CK (11 for laboratory and 9 for field-based vs. 6 and 6) compared to low MQI. A prior MQI screening can provide feedback to understand the magnitude response. Individual responsiveness should be taken into consideration for maximizing eccentric exercise prescription.

## 1. Introduction

Aging is a multifactorial process characterized by detrimental changes in several cells and tissues that represent significant contributors to a progressive decrease in physiological function, physical frailty, and mortality [1]. The detrimental effect of aging on the musculoskeletal system is linked with fall risk, gait disorders, functional disability, and low muscle quality index (MQI) [2,3]. This is relevant, as the growing number of older persons is increasing faster than any other age group, and by 2050, individuals 60 years or older are expected to comprise 56% of the global adult population [4].

Muscle quality index refers to the tissues capacity to perform functions such as contraction, metabolism, and electrical conduction [3]. Various methods to assess MQI in both laboratory and field-based research have been reported. In clinical research, MQI can be measured as handgrip strength or leg strength normalized to muscle mass determined by dual-energy X-ray (DXA) or computed tomography [3]. In field-based practice, strength is adjusted for body mass index (BMI) or body mass [5,6]. Low MQI is considered a strong predictor of lower extremity function, history of falls, and skeletal muscle fatigue. Older individuals with lower MQI display a greater risk of hospitalization, poor glycemic control, and higher intramuscular lipid infiltration than those with a higher MQI [7,8]. Moreover, evidence points that MQI is a promising concept to describe intramuscular changes that occur during aging, as it has been shown to be a stronger predictor of muscle performance than strength, muscle thickness, or body composition alone [9]. Hence, MQI might be a crucial clinical and practical tool to identify older adults at the greatest risk for developing future physical disability.

Recent observations provide evidence that planned and structured resistance exercise is largely recommended as an efficient, nonpharmacological intervention that increases muscle quality in obese and non-obese older adults [10,11]. The most recent recommendations from the National Strength and Conditioning Association highlights the importance of eccentric exercise to improve muscle mass, physical function, and power in older individuals with limited tolerability and diminished muscular strength [10,12,13]. Eccentric resistance exercise has gained considerable attention due to its unique properties, such as low energy cost and high muscle force production compared to concentric action [14,15]. However, the acute physiological response to eccentric exercise in sedentary obese older women with a different MQI remains unclear.

Some studies demonstrate that eccentric exercise resulted in increased interleukin 6 (IL-6) and creatine kinase (CK) levels that returned to pre-exercise values within hours [16,17]. Such transient effects may be important in modulating muscle regeneration, anti-inflammatory effects, and energy homeostasis, as well as facilitating extracellular glucose uptake and free fatty acid mobilization during exercise, which has relevancy for aging [18,19]. However, an exercise involving a significant muscle mass and type II fibers recruitment is necessary to produce a marked systemic IL-6 and CK response [20,21], which indirectly suggests that MQI may modulate these molecules. A greater rise in IL-6 and CK activity post-exercise may be related to greater force-generation per fiber during muscle contractions and cellular energy availability [21]. In addition, since contracting skeletal muscle per se is an important source of IL-6 and CK found in the blood circulation [20,21], a limited MQI may be insufficient to increase plasma IL-6 and CK above pre-exercise level. Therefore, prior MQI screening may have important practical applications on exercise prescription and monitoring because it can clarify adaptive responses, as well as determine specific strategies in sedentary obese older women.

Mann et al. [22] proposed baseline muscle phenotypes as a critical factor contributing to individual variation in response to acute exercise. They hypothesized that the relationship between baseline parameters and subsequent training responses may be related to the capacity for improvement [22]. The substantial interindividual variations in response to specific exercise doses might clarify valuable insights into the adaptations caused by eccentric exercise, thus promoting health. Moreover, it might provide useful approaches to coaches to make exercise session adjustments, optimizing outcomes.

Considering that exercise-induced IL-6 and CK levels depend on muscle mass involved and force production [23], understanding the role of distinct MQI on IL-6 and CK responses following acute eccentric resistance exercise in sedentary obese older women is an essential first step in describing potential adaptive mechanisms. Advances that have occurred in molecular components in response to eccentric exercise could provide fascinating insights for practical diagnostic and/or therapeutic fields related to musculoskeletal health. Thus, the purpose of this study was to evaluate the time-course responses on IL-6 and CK levels following acute eccentric resistance exercise (pre, 0, 3, 24 and 48 h postexercise) in sedentary obese older women with different MQI values. We secondarily evaluated the responsiveness of the molecules based on the minimal clinical important difference (MCID). We hypothesized that sedentary obese older women with high MQI would display distinct circulating IL-6 and CK levels in response to acute eccentric exercise with a higher proportion of high responders for IL-6 and CK when compared to the low MQI group.

## 2. Materials and Methods

### 2.1. Participants

Eighty-eight older women from Brasilia (Brazil) were obtained for analysis. The individuals were recruited through local advertisements. Participants were divided into two groups: high MQI and low MQI according to the 50th percentile. The high MQI was defined as muscle quality ≥4.73 for laboratory and ≥1.79 for field-based MQI. No clinical or physiological criteria have been established in the literature to define the cutoff value of percentile. Hence, initially we performed an exploratory analysis to determine optimal contrast between groups. Stratifying into halved displayed a stronger contrast between groups when compared to thirds or fourths.

Traits of older women are reported in Table 1 and Table 2. Females volunteers were included if they were aged ≥60 years, were in sedentary condition, and had body fat percentage >30%, besides full anthropometric measurements. Obesity condition was confirmed through of the advices of the National Institute of Diabetes [24], presuming a 30% cut-off point. Subjects were categorized as a sedentary person according to the International Physical Activity Form. Older women with autoimmune diseases or use of medications (anti-inflammatory drugs, hormone therapy, and all beta blockers groups) that could bias the trials were withdrawn from the study.

The research was approved by the local Institutional Research Ethics Committee (protocol 59071116.8.3001.5553/2017). The study design and employed procedures were in accordance with ethical guidelines and the Declaration of Helsinki. Each participant was fully informed in relation to the risks associated with study involvement and gave their written informed consent.

### 2.2. Muscle Strength Evaluation

Muscle strength was assessed by the ten repetitions maximum test (10 RM) consistent with prior investigation [25]. The participants visited the laboratory two times. On the initial visit, older women completed a physical activity form, anthropometric procedures, dual-energy X-ray absorptiometry (DXA) and accomplished familiarization of a leg extension movement (Righetto, Sao Paulo, Brazil), with 3 sets of 8–10 submaximal repetitions. Thereafter, they performed the 10 RM test. Participants rested three days and completed the 10 RM test again to verify reproducibility (R = 0.99). The 10 RM test was finished when the older women were unable to complete the optimal movement or when concentric failure happened. Rest intervals between sets of the 10 RM test were 3 min. The volunteers refrained from caffeine and alcohol, and they did not perform exercise in the prior week of the examinations. The muscle strength evaluation was scheduled between 2:00 and 4:00 p.m. at controlled room temperature. The leg extension exercise was selected since lower limb strength has an association with dependency in older adults [26].

Seven days after the 10 RM tests, participants finished an eccentric resistance exercise (ERE) session as previously described [17]. Upon arrival, participants began with a cycle ergometer for 10 min at 60 rpm and 50 W, followed by 10 leg extension repetitions at 50% of the 10 RM with rest intervals of 3 min. The ERE was achieved on the bilateral knee extension machine with a load equivalent of 110% of the 10 RM. Participants performed only the eccentric phase of the lift (lowering for 2–3 s); at the end of eccentric action, the investigator moved the load through the concentric portion of the range of motion to begin the subsequent eccentric action. Participants completed seven sets of 10 repetitions with a rest of 3 min between sets. From the 10 RM results, the 1 RM value for each participant was estimated based on an equation from prior research [27].

### 2.3. Muscle Quality Index

Laboratory MQI used the ratio of predicted 1 RM leg extension to the entire lower limb muscle in kilograms measured by DXA. Field-Based MQI used the ratio of predicted 1 RM leg extension divided by the BMI. The validity and reliability of the MQI measures (field-based and laboratory) have been previously reported, and the definition of muscle quality adopted in this study is commonly used in large-scale studies because of its convenience [6,28,29,30,31,32].

### 2.4. Body Composition

Body fat percentage and fat-free mass were established by DXA (General Electric-GE model 8548 BX1 L, year 2005, Lunar DPX type, software Encore 2005; Rommelsdorf, Germany). DXA calibration was provided, and phantom was utilized to check calibration daily before evaluation. The assessments contained a complete body scan of the older women in the supine position, with the apparatus always adjusted and conducted by a specialist. Participants were ordered to remove any metal accessories prior to lying in the supine position. The legs were secured by nonelastic straps at the knees and ankles, and the arms were aligned along the trunk with the palms facing the thighs. No exercise, smoking, alcohol, or diuretic-enhancing products were allowed 48 h prior to examination, and participants were oriented to maintain habitual dietary intake and water ingestion.

### 2.5. Blood Biomarkers

Older women checked into the research laboratory between 08:00–11:00 a.m. Later, an overnight fast (14 h), and blood samples of 15 mL were drawn from the antecubital vein into standard tubes (Becton Dickinson, São Paulo, Brazil) using standard aseptic techniques. All blood samples were centrifuged at room temperature at 2500 rpm for 15 min. The serum fraction was stored in 1.5 mL aliquots at −80 °C. Lipid profile, urea (Ureasi-GLDH) and uric acid were evaluated by the enzymatic colorimetric method on Autohumalyzer apparatus (Human GMBH, Wiesbaden, Germany). High-density lipoprotein cholesterol (HDL-C) was established by ionic exchange by colorimetric reaction with Linco^®^ Research Inc. kit (St Louis, MO, USA), and blood glucose levels were determined by hexokinase enzymatic method.

**Figure 1:**
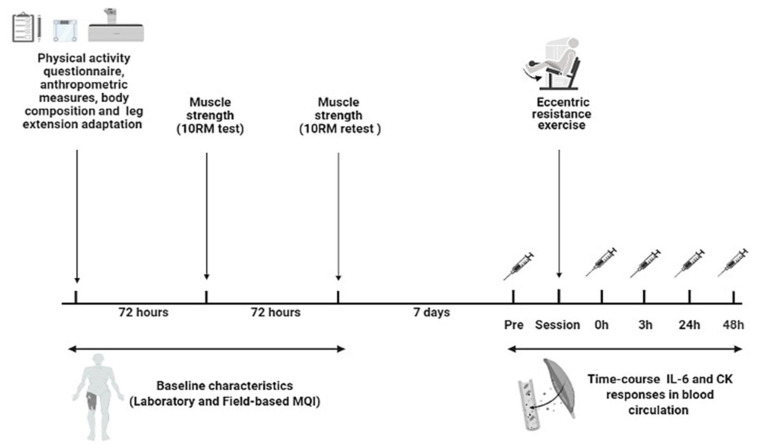
Schematic study design and timeline used to examine the time course of interleukin-6 (IL-6) and creatine kinase (CK) levels following acute eccentric resistance exercise in sedentary obese older women with different muscle quality index (MQI).

IL-6 levels were assessed by Quantikine high sensitivity commercial enzyme-linked immunosorbent assay Kit (R&D Systems, Minneapolis, MN, USA). The intra-assay coefficient of variation of the kit was 1.5–5.6% for cytokine and 4.3–6.4% for CK. The CK levels were concluded by application of a Reflotron CK analyze using the Reflotron system (Boehringer Mannheim GmbH, Mannheim, Germany). The CK and IL-6 evaluations were realized in triplicate. For field MQI, IL-6 was barely detectable for 14 participants in the high MQI and 21 participants in the low MQI group. For laboratory MQI, IL-6 was barely detectable for 16 participants in the high MQI and 19 participants in the low MQI group. The schematic study design and timeline is shown in Figure 1.

### 2.6. Statistical Analysis

A two-way mixed ANOVA was used to compare the mean differences between groups (laboratory MQI and field-based MQI) on IL-6 and CK levels. Considering the non-normal distribution of IL-6 and CK, the concentrations were log transformed to derive a near-normal distribution, and a constant of 1 was added before log transformation for IL-6. In addition, corrections based on Greenhouse–Geisser were used, as sphericity was violated. For the other non-parametric variables, a chi-square for proportions and Mann–Whitney test for comparisons of the medians between groups were used. When expected cell counts were less than five, Fisher’s exact test (2 × c) was applied for chi-squared test. For the parametric data, an unpaired Student’s *t* test was used to verify possible differences between means (participant characteristics and area under the curve (AUC)). For the effect size, the following conventions were adopted: *f* = 0.10 (small), *f* = 0.25 (medium) and *f* = 0.40 (large) [33]. An alpha level of ≤0.05 was considered significant. All analyses were conducted with SPSS software version 18.0 (SPSS Inc., Chicago, IL, USA). In addition, Graphpad Prism 6.0 software was also used (San Diego, CA, USA).

In addition, the minimal clinical important difference (MCID) was calculated, and 0.80 (large effect) was multiplied by the standard deviation of the pre-exercise values of IL-6 levels and CK levels [34,35,36]. Thereby, the MCID for field-based IL-6 and laboratory IL-6 was 2.44 pg/mL and for CK was 36.87 U/L. Based on previous evidence, to be considered a responder, the participant was required to present increments in IL-6 of ≥2.44 pg/mL (between pre- and post-exercise 3 h) [37]. For CK, to be considered a responder, the participant was required to present increments in CK of ≥6.87 U/L (between pre- and post-exercise 48 h) [38]. High responders were classified as increases in IL-6 levels of ≥2.44 pg/mL, and low responders were classified as declines in IL-6 levels of ≥−2.44 pg/mL. For CK, high responders were classified as CK level increments of ≥ 36.87 pg/mL, and low responders were classified as declines in CK levels ≥−36.87 pg/mL.

## 3. Results

### 3.1. Participants Characteristics

For the laboratory muscle quality participant characteristics, the low MQI group displayed a lower laboratory MQI, 10 RM leg extension and 1 RM leg extension strength when compared to the high MQI group. In addition, the low MQI group presented higher glycemia when compared to the high MQI group (Table 1).

For the field-based muscle quality participants’ characteristics, the low MQI group displayed a lower field MQI, 10 RM leg extension and 1 RM leg extension strength when compared to the high MQI group. In addition, the low MQI group presented higher glycemia, higher body mass index and a higher percentage of body fat when compared to the high MQI group (Table 2). In addition, there was a statistically significant association between MQI and the presence of type II diabetes mellitus, and the high MQI had no participants with type II diabetes mellitus (Table 2). Despite the differences observed between groups for obesity indexes, there was no relationship between BMI or body fat % and the dependent variables (IL-6 and CK baseline levels) as assessed by visual inspection of the scatterplot. Thus, if the relationships were not linear, the assumption of analysis of covariance (ANCOVA) was not suitable to proceed for time-course effects in eccentric exercise. There was a no correlation between BMI and IL-6 (r = −0.113; *p* = 0.382) or CK levels (r = 0.195; *p* = 0.069). Similarly, there was a no correlation between body fat % and IL-6 (r = −0.136; *p* = 0.292) or CK levels (r = 0.180; *p* = 0.093).

### 3.2. Time-Course Effects of IL-6 and CK Levels in Low and High MQI

**Figure 2:**
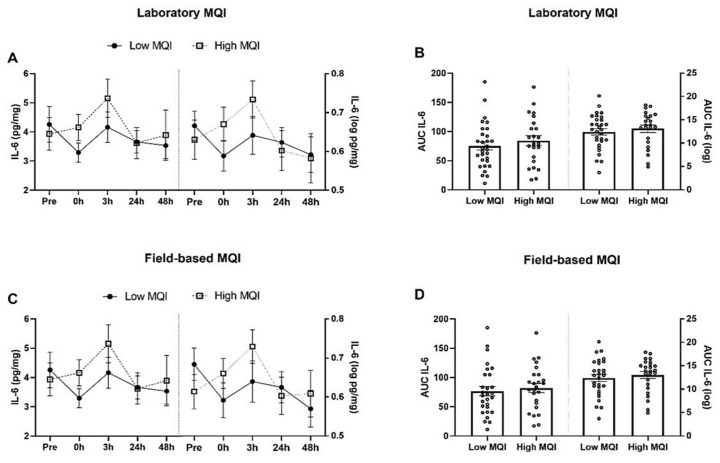
Interleukin 6 (IL-6) levels in blood circulation (mean ± SE) after an acute bout of eccentric exercise in older obese women with low and high muscle quality. (**A**) Laboratory IL-6 levels; (**B**) AUC laboratory IL-6 levels; (**C**) field-based IL-6 levels; (**D**) AUC field-based IL-6 levels.

There was no statistically significant interaction between groups and time on laboratory IL-6 levels, F (2.62, 133.64) = 2.05, *p* = 0.117 (Figure 2A) and field-based IL-6 levels, F (2.62, 134.05) = 1.701, *p* = 0.176 (Figure 2C). In addition, there were no changes in quantification of the AUC (*p* > 0.05); Figure 2B,D).

**Figure 3:**
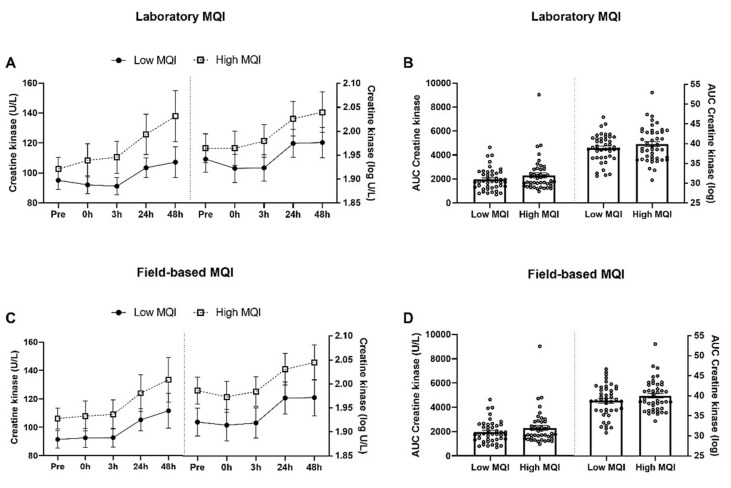
Creatine kinase (CK) levels in blood circulation (mean ± SE) after an acute bout of eccentric exercise in older obese women with low and high muscle quality. (**A**) Laboratory CK levels; (**B**) AUC laboratory CK levels; (**C**) field-based CK levels; (**D**) AUC field-based CK levels.

There were no statistically significant interactions between groups and time on laboratory CK levels, F (3.14, 270.11) = 0.064, *p* = 0.982 (Figure 3A), and field-based CK levels, F (2.14, 184.05) = 1.116, *p* = 0.333 (Figure 3C). Moreover, there were no changes in quantification of the AUC (*p* > 0.05; Figure 3B,D).

### 3.3. Responsiveness for IL-6 Levels Based on MCID

**Figure 4:**
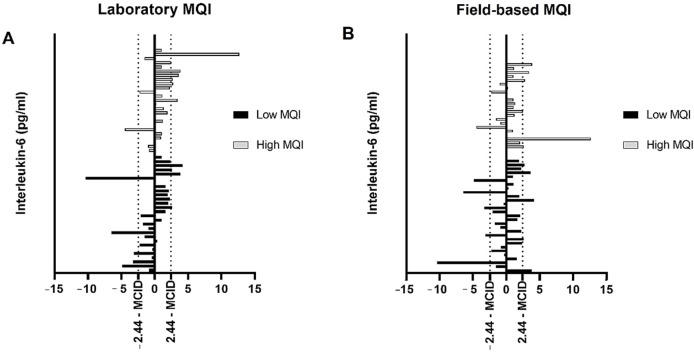
Responsiveness for the interleukin 6 (IL-6) levels based on minimal clinical important difference (MCID). (**A**) Laboratory IL-6 levels; (**B**) field-based IL-6 levels.

For the responsiveness based on MCID for the low MQI group, four (14.28%) and five (16.66%) responders exceeded MCID for IL-6 in the laboratory and field-based analysis, respectively (Figure 4). However, five (16.66%) participants displayed a low response in the laboratory analysis, while four (14.28%) displayed a low response in the field-based analysis (Figure 4). Thirty-six (69.06%) individuals show an unchanged response (“non-responders”) after exercise for laboratory and field-based analysis.

For the high MQI group, seven (28.0%) and six (26.08%) responders exceeded MCID for IL-6 in the laboratory and field-based analysis, respectively (Figure 4). One (4%) participant displayed a low response in the laboratory analysis, while two (8.69%) participants displayed a low response in the field analysis (Figure 4). Seventeen (68.0%) and fifteen (65.23%) individuals showed an unchanged response (“non-responders”) after exercise for laboratory and field-based analysis, respectively.

### 3.4. Responsiveness for CK Levels Based on MCID

**Figure 5:**
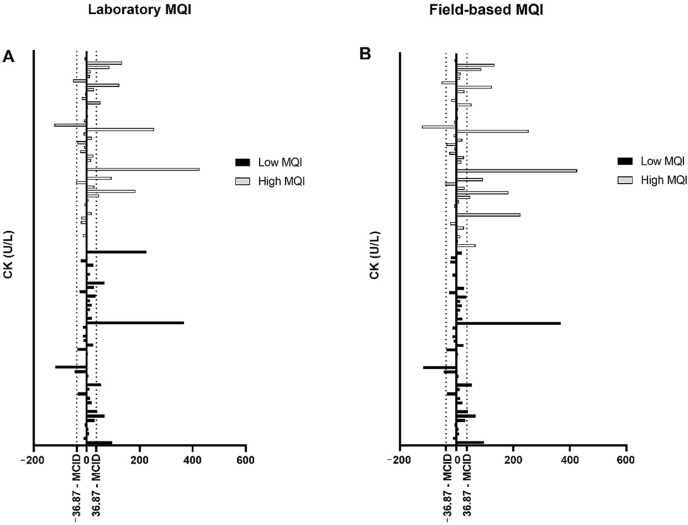
Responsiveness for creatine kinase (CK) levels based on minimal clinical important difference (MCID). (**A**) Laboratory CK levels; (**B**) field-based CK levels.

For the responsiveness based on MCID for the low MQI group, six (13.63%) and six (13.63%) responders exceeded MCID for CK in the laboratory and field-based estimate of MQI, respectively (Figure 5). Two (4.54%) participants displayed a low response in the laboratory analysis, while two (4.54%) participants displayed a low response in the field-based estimate (Figure 5). Thirty-six (81.83%) individuals show an unchanged response (“non-responders”) after exercise for laboratory and field-based analysis.

For high MQI group, nine (20.45%) and eleven (25.0%) responders exceeded MCID for CK in the laboratory and field-based estimate of MQI, respectively (Figure 5). Three (6.81%) participants displayed a low response in the laboratory analysis, and three (6.81%) participants displayed a low response in the field-based analysis (Figure 5). Thirty-two (72.74%) and thirty (68.19%) individuals show an unchanged response (“non-responders”) after exercise for laboratory and field-based analysis, respectively.

### 3.5. Effect Size

Considering the effect size values between groups for the main effect of time, both groups displayed small magnitudes (Table 3).

## 4. Discussion

**Figure 6:**
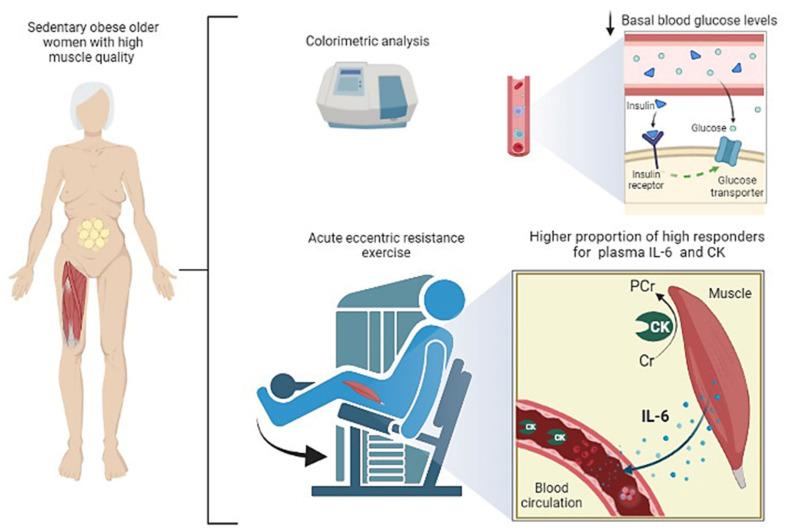
Overview of main findings and hypothetical mechanism involved in high MQI group.

The current study provides several new findings: (1) Acute eccentric exercise in sedentary obese older women with different MQI does not impact the IL-6 and CK post-exercise response. (2) The high MQI group displayed a lower proportion of low responders and a higher proportion of high responders for IL-6 and CK when compared to the low MQI group. (3) The low MQI group displayed higher glycemia levels as compared with the high MQI group. (4) The field-based MQI displayed an association with diabetes mellitus (Figure 6). The present study adds to the growing body of data showing that baseline muscle quality modulates individual responsiveness of important molecules following acute eccentric exercise and may be a key phenotype in adaptive responses.

Skeletal muscle has recently been known as an secretory organ, which has different mechanical and metabolic functions [39]. This tissue accounts for more than 80% of insulin-stimulated glucose uptake and has a major impact on whole-body metabolic homeostasis, and it is the main factor for diabetes mellitus development [40], suggesting that it could be relevant for the aging process. In the current study, the low MQI group presented a higher glycemic level when compared to the high MQI group, potentially highlighting the importance of muscle strength and hypertrophy in ameliorating metabolic dysfunction.

Similar to how a healthy muscle phenotype seems to promote beneficial metabolic adjustments, lower strength and mass seem to impair it. The muscle tissue could be a key response to explain the association between MQI and diabetes mellitus found in this study. Regarding plausible mechanisms, muscle tissue can promote improved glucose control by AMPK-IRS1 axis activation, increasing the concentration of GLUT-4 translocation [41,42]. Moreover, decreased insulin sensitivity patterns are linked to a more glycolytic fiber type profile, which is a common adaptation during muscle strength gains and hypertrophic processes [43].

Although other studies have found increased systemic IL-6 levels after an acute eccentric exercise bout in older adults [16,44], we observed that different MQIs do not impact the IL-6 time-course response. A possible explanation is that myocytes and immune cells primarily produce IL-6 release in the skeletal muscle interstitium due to the remodeling process, biological activities and metabolic cell demands [45,46], which obscure prominent bloodstream responses. Notwithstanding, substantial changes in circulating IL-6 levels after muscle contraction would have been more evident in serum than in plasma since cytokines appear to be released primarily in this blood fraction [37,47]. Further, the complexity and interindividual variability between sedentary obese older women in our sample may have contributed to our IL-6 observations. Additionally, the biochemical kit, antibody type, and sample storage time vary extensively and may affect comparisons with previous investigations [16,44,48].

Windsor et al. [49] reported that there was no difference in the acute IL-6 responses to a bout of exercise between older adults with lower and higher levels of cardiorespiratory fitness and physical activity. These findings support the suggestion that for older adults, acute cytokine responses to exercise may be reduced [49]. However, this prior study did not include individuals with high and low MQI, which obscures the fundamental role of skeletal muscle on acute IL-6 responses to exercise. Muñoz-Cánoves et al. [50] clarified that IL-6 signaling, produced locally by different cell types, positively impacts the proliferative capacity of muscle stem cells [50]. Thus, the compensatory physiological mechanism to provide enough muscle progenitors in conditions involving a high cell number, such as muscle regeneration processes and hypertrophic growth after acute eccentric exercise, can explain why the high MQI group displayed a higher proportion of high responders. Moreover, a greater response in IL-6 post-exercise observed in the high MQI group may be related to an adaptive process for modulating energy homeostasis during greater force-generation [20]. It has been shown that increases in IL-6 levels provoked by acute exercise could have the advantage to modulate the lipolysis process accompanied by anti-inflammatory effects [51]. Furthermore, IL-6 can stimulate alternative M2 macrophage activation, which is implicated in the protection from obesity-induced chronic inflammatory systemic and insulin resistance [52], reinforcing the importance of our findings.

In a prior study, Tajra et al. [17] identified a high responder group for IL-6 in obese older women following acute eccentric exercise. This group exhibited significantly greater IL-6 concentrations at 0 and 24 h when compared to the non-responder group. However, the authors did not evaluate baseline characteristics that are potentially associated with these responses [17]. Considering that the high MQI group displayed a lower proportion of low responders when compared to the low MQI group, a less healthy muscle phenotype seems to allow for a smaller window for systemic IL-6 release. Thus, this post hoc analysis represents an essential first step in understanding MQI as a promising baseline phenotype to explain the possibility of blunted IL-6 response to eccentric exercise. However, a range of blood markers likely plays a role in an individual’s response to intervention and should be examined in future investigations.

Conversely, exercise-induced muscle damage increases cell membrane permeability to skeletal muscle enzymes, initiating leakage of multiple metabolic molecules and cytokines in the bloodstream [53]. Although it has been established that transient increases in CK and IL-6 levels immediately following eccentric exercise are expected, chronically high levels are not desirable [54]. An important finding of the present study was that we observed similar baseline CK and IL-6 levels between groups, but the high MQI, displayed a higher proportion of responders post-acute exercise when compared to the low MQI group. Consistent with previous studies, high MQI may be more susceptible to damage, as they must generate a greater force per fiber during eccentric contractions [21,55], which clarifies individual responsiveness observed in this current investigation.

Hence, different basal muscle qualities may be responsible for the different responsiveness proportions between groups represented in the current study. Considering the acute intervention and proposal of this study, comparisons with other studies are difficult. High levels of tumor necrosis factor alpha (TNF-α) may disrupt the regeneration of muscle tissue, and a previous study by Fisher [56] demonstrated that older women who responded to gains in fat-free mass after 16 weeks of resistance training displayed lower TNF-α levels than older women who did not respond to gains in fat-free mass. In addition, older women, who were considered responders to gains in fat-free mass, showed a lower basal IL-6 level after 16 weeks post-training when compared to non-responder participants. Besides, field-based MQI between groups following training showed a higher MQI in responders than in non-responders (1.43 vs. 1.30). Furthermore, reductions in CRP levels are related to increases in skeletal muscle mass, which reinforces that muscle phenotype can modulate the capacity for improvement in training responses [57].

In an animal model, obese mice showed greater myofiber membrane disruption after eccentric exercise compared to lean animals, which consequently could indirectly indicate higher CK levels [58]. Similarly, healthy male participants responsive for CK levels during acute eccentric resistance exercise (2 sets of 25 contractions of the elbow flexors) demonstrated higher baseline body fat compared to the low responder group [59]. However, in the present study, the low field-based MQI group displayed a higher body fat percentage when compared to the high MQI group, but they had the same CK time-course responses after eccentric exercise. Further investigations are required to explain this contradictory result, and it may suggest that other adjacent molecular pathways or post-translational regulation processes were involved in producing the observed CK levels. Although CK and IL-6 levels are associated with muscular microtrauma, CK responsiveness may not necessarily be related to inflammatory response following acute eccentric exercise [17].

Our findings indicate lower CK levels, compared to other studies that used an acute eccentric protocol [16,59,60], which can indicate that our protocol may provide an adequate training stimulus with a lower stress level to the participants. However, as this study was acute, a longitudinal investigation would be warranted to support the aforementioned. Furthermore, most studies still report biological responses to eccentric exercise as a group average, and there are scarce data regarding which individual baseline characteristic or muscle phenotype elicits a more robust effect on the release of myokines or proteins/hormones [16,44,61,62]. Moreover, the magnitude of the metabolic demand and fatigue may directly affect the cytokine and CK responses to acute eccentric exercise [62]. Considering the effect of MQI on IL-6 and CK response, a previous study stated that responsiveness might not be a common occurrence but the result of inappropriate statistical analyses [63]. Furthermore, responsiveness might depend on hereditary factors, baseline phenotype, readiness to train, training status, sleep and stress, and nutritional status [22].

One of the essential aspects of the practical application is the importance to analyze MQI values before applying an exercise session to optimize results and understand the dose–response relationship. The older women with low MQI may be offered guidance for reducing potential consequences of low responses or increasing benefits through exercise by changing the prescription according to their individual responsiveness to training. Here, we employed a method that multiplied the pre-exercise standard deviations of IL-6 and CK levels by a large effect size (0.8) to obtain our threshold for high responders since no evidence-based MCID has been reported [34,35,36]. However, the MCID for IL-6 in this study is different from a previous study (3.21 pg/mL for IL-6) that evaluated the acute effect of other protocols (high velocity vs. traditional resistance training) on cytokine response [64]. Furthermore, a previous study showed that older adults in the lowest quartile of MQI presented a higher risk of self-reported mobility function and low functional capacity test scores for chair tests and gait speed compared to the highest quartile of MQI [28]. However, we also used the 20th percentile in our analyses and found no differences between groups. Therefore, additional randomized controlled trials that establish a threshold of clinically relevant changes on IL-6 and CK levels in older women with different MQI values are necessary. These investigations can be important to define physiological criteria on this topic.

This study has some limitations worth mentioning. We did not include a non-obese group or investigate genetic variability between the participants. Skeletal muscle biopsy would be necessary to examine mechanisms at a cellular level. Finally, our results are limited to a time frame. Thus, whether longer periods of training could change muscle quality and/or influence IL-6/CK responsiveness remains a provocative hypothesis for further investigation.

## 5. Conclusions

In summary, different muscle quality indexes do not appear to impact the time course of IL-6 and CK response following an acute eccentric resistance exercise session. However, there was considerable inter-individual variability in the responses of IL-6 and CK as demonstrated by MCID. The high MQI group displayed a higher proportion of high responders for IL-6 and CK when compared to the low MQI group. It is possible that responsiveness may contribute to the mediation of health effects and should be taken into consideration for maximizing benefits in the context of personalized eccentric exercise prescription. A prior MQI screening might potentially provide important feedback to exercise physiologists, clinicians, and coaches to understand the magnitude response and consequently adaptive effects inherent to eccentric exercise in sedentary obese older women.

## Figures and Tables

**Table 1:** Laboratory muscle quality participants characteristics.

	Low MQI *n* = 44		High MQI *n* = 44		*p*
Age, Years	68.55 ± 6.20		70.41 ± 5.92		0.51
Body mass index, kg/m^2^	27.71 ± 5.03		27.75 ± 4.52		0.97
Body fat, %	41.47 ± 6.16		41.42 ± 5.18		0.96
Upper body muscle mass, kg	3.71 ± 0.62		3.87 ± 0.56		0.21
Lower body muscle mass, kg	11.04 ± 1.40		11.17 ± 1.28		0.64
Appendicular muscle mass, kg	14.75 ± 1.91		15.04 ± 1.60		0.44
Body mass, kg	64.44 ± 12.85		64.95 ± 11.47		0.84
Height, m	1.52 ± 0.06		1.52 ± 0.05		0.69
Laboratory muscle quality index	3.64 ± 0.60		5.16 ± 0.64 *		0.001
10 RM leg extension, kg	30.19 ± 6.19		43.20 ± 6.78 *		0.001
1 RM leg extension, kg	40.27 ± 8.26		57.62 ± 9.04 *		0.001
Creatine kinase, U/L	91.52 ± 41.86		106.18 ± 49.32		0.13
Total cholesterol, mg·dL^−1^	203.18 ± 31.06		210.57 ± 45.88		0.37
Triglycerides, mg·dL^−1^	144.09 ± 68.92		142.33 ± 72.58		0.90
High-density lipoprotein, mg·dL^−1^	46.95 ± 10.14		48.83 ± 12.69		0.44
Low-density lipoprotein, mg·dL^−1^	127.41 ± 28.25		136.18 ± 36.21		0.20
Very low-density lipoprotein, mg·dL^−1^	28.81 ± 13.80		27.19 ± 11.08		0.54
Uric acid, mg·dL^−1^	4.50 ± 1.28		4.79 ± 1.96		0.41
Glycemia, mg·dL^−1^	103.72 ± 37.05		91.60 ± 10.47 *		0.04
Urea, mg·dL^−1^	34.67 ± 7.87		35.73 ± 10.70		0.59
Interleukin-6, pg/mL	4.48 ± 3.44		4.61 ± 7.48		0.93
Creatine Kinase, U/L	91.52 ± 41.89		106.18 ± 49.32		0.13
	Low MQI		High MQI		*p*
	Yes	No	Yes	No	
Hypertension, %	26.1	23.9	28.4	21.6	0.83
Diabetes, %	5.7	44.3	1.1	48.9	0.20

Note: Values are expressed as mean (standard deviation) or percentage. MQI, muscle quality index; * *p* < 0.05 low MQI vs. high MQI.

**Table 2:** Field muscle quality participant characteristics.

	Low MQI *n* = 44		High MQI *n* = 44		*p*
Age, Years	69.05 ± 6.10		69.91 ± 6.14		0.510
Body mass index, kg/m^2^	29.22 ± 4.98		26.24 ± 4.06 *		0.003
Body fat, %	43.02 ± 5.61		39.87 ± 5.31 *		0.008
Upper body muscle mass, kg	3.76 ± 0.65		3.81 ± 0.53		0.70
Lower body muscle mass, kg	11.01 ± 1.36		11.20 ± 1.32		0.51
Appendicular muscle mass, kg	14.78 ± 1.86		15.02 ± 1.66		0.53
Body mass, kg	67.20 ± 13.48		62.18 ± 10.10		0.051
Height, m	1.51 ± 0.06		1.53 ± 0.05		0.052
Field-based muscle quality index	1.42 ± 0.25		2.16 ± 0.33 *		0.001
10 RM leg extension, kg	31.05 ± 6.78		42.34 ± 7.73 *		0.001
1 RM leg extension, kg	41.41 ± 9.04		56.47 ± 10.31 *		0.001
Creatine kinase, U/L	95.07 ± 39.82		102.64 ± 51.79		0.44
Total cholesterol, mg·dL^−1^	203.04 ± 31.64		210.70 ± 45.46		0.36
Triglycerides, mg·dL^−1^	138.93 ± 51.59		147.49 ± 85.55		0.57
High-density lipoprotein, mg·dL^−1^	47.90 ± 11.05		48.87 ± 11.98		0.99
Low-density lipoprotein, mg·dL^−1^	127.35 ± 30.25		136.23 ± 34.53		0.20
Very low-density lipoprotein, mg·dL^−1^	27.77 ± 10.31		28.23 ± 14.43		0.86
Uric acid, mg·dL^−1^	4.76 ± 1.34		4.52 ± 1.93		0.50
Glycemia, mg·dL^−1^	103.29 ± 36.73		91.03 ± 10.88 *		0.024
Urea, mg·dL^−1^	35.24 ± 6.77		35.15 ± 11.46		0.96
Interleukin-6, pg/mL	4.13 ± 3.36		5.02 ± 7.70		0.44
Creatine Kinase, U/L	95.07 ± 39.82		102.64 ± 51.79		0.44
	Low MQI		High MQI		*p*
	Yes	No	Yes	No	
Hypertension, %	27.3	22.7	20.5	29.5	0.28
Diabetes, %	6.8	43.2	0.0	50	0.026

Note: Values are expressed as mean (standard deviation) or percentage. MQI, muscle quality index; * *p* < 0.05 Low MQI vs. High MQI.

**Table 3:** Values of effect size for main effect of time.

	LMQI	HMQI
**Parameters**	*f*	*f*
**Laboratory MQI**		
IL-6, pg/ml	0.073 (small)	0.089 (small)
CK, U/L	0.070 (small)	0.076 (small)
**Field-based MQI**		
IL-6, pg/ml	0.044 (small)	0.104 (small)
CK, U/L	0.061 (small)	0.091 (small)

Note: *f*, effect size; IL-6, interleukin 6; LMQI, low muscle quality index; HMQI, high muscle quality index.

## Data Availability

The datasets used and/or analyzed during the current study will be made available by the corresponding author upon reasonable request.
